# Regulation of RKIP Function by *Helicobacter pylori* in Gastric Cancer

**DOI:** 10.1371/journal.pone.0037819

**Published:** 2012-05-25

**Authors:** Erika L. Moen, Sicheng Wen, Talha Anwar, Sam Cross-Knorr, Kate Brilliant, Faith Birnbaum, Sherida Rahaman, John M. Sedivy, Steven F. Moss, Devasis Chatterjee

**Affiliations:** 1 Department of Medicine, Rhode Island Hospital, Providence, Rhode Island, United States of America; 2 Centers of Biomedical Research Excellence (COBRE), Rhode Island Hospital, Providence, Rhode Island, United States of America; 3 Department of Pathobiology Graduate Program, Brown Univesity, Providence, Rhode Island, United States of America; 4 Department of Molecular Biology, Cell Biology and Biochemistry, Brown University, Providence, Rhode Island, United States of America; Veterans Affairs Medical Center (111D), United States of America

## Abstract

*Helicobacter pylori (H. pylori)* is a gram-negative, spiral-shaped bacterium that infects more than half of the world’s population and is a major cause of gastric adenocarcinoma. The mechanisms that link *H. pylori* infection to gastric carcinogenesis are not well understood. In the present study, we report that the Raf-kinase inhibitor protein (RKIP) has a role in the induction of apoptosis by *H. pylori* in gastric epithelial cells. Western blot and luciferase transcription reporter assays demonstrate that the pathogenicity island of *H. pylori* rapidly phosphorylates RKIP, which then localizes to the nucleus where it activates its own transcription and induces apoptosis. Forced overexpression of RKIP enhances apoptosis in *H. pylori*-infected cells, whereas RKIP RNA inhibition suppresses the induction of apoptosis by *H. pylori* infection. While inducing the phosphorylation of RKIP, *H. pylori* simultaneously targets non-phosphorylated RKIP for proteasome-mediated degradation. The increase in RKIP transcription and phosphorylation is abrogated by mutating RKIP serine 153 to valine, demonstrating that regulation of RKIP activity by *H. pylori* is dependent upon RKIP’s S153 residue. In addition, *H. pylori* infection increases the expression of Snail, a transcriptional repressor of RKIP. Our results suggest that *H. pylori* utilizes a tumor suppressor protein, RKIP, to promote apoptosis in gastric cancer cells.

## Introduction

Gastric cancer is the fourth most frequently diagnosed malignancy in the world. In 2007, approximately one million new gastric cancer cases leading to approximately 800,000 deaths worldwide were recorded, making it the second most common cause of death from cancer [1]. Gastric cancer is currently the seventh leading cause of cancer deaths in the US, with approximately 21,500 new cases diagnosed in 2011 (http://www.cancer.gov/cancertopics/types/stomach). The gram-negative, spiral shaped bacterium *Helicobacter pylori (H. pylori)* infects more than half of the world’s population and has been identified as a major risk factor in gastric carcinogenesis [2]. The World Health Organization and the International Agency for Research on Cancer designated it as a class I carcinogen in 1994 [3]. Our current understanding of *H. pylori*-induced carcinogenesis is that the bacterium and the associated chronic inflammatory response promote gastric epithelial cell death by apoptosis [4], with subsequent hyper-proliferation [5], and free radical production [6] all of which contribute to a slow and progressive sequence of changes in the gastric mucosa that ultimately favor progression towards cancer. This model is consistent with reports that pro-inflammatory cytokine gene polymorphisms that increase the intensity of the inflammatory response are related to increased gastric cancer risk [7].

*H. pylori* adheres closely to gastric epithelial cells and can induce apoptosis directly [8]. The cag (cytotoxic-associated gene) pathogenicity island (cag PAI) of *H. pylori* is a 40 kB segment of DNA that contains genes encoding for components of a type IV bacterial secretion system [9]. Within this region is the *cagA* gene which encodes CagA, an immunodominant protein of 121–145 kDa [9]. *H. pylori* strains possessing and expressing the cag PAI are more often associated with peptic ulcer disease and gastric cancer in Western populations than strains that do not [9]. Upon its injection via the type IV secretion system into host gastric epithelial cells, CagA may subsequently become phosphorylated by Src-family tyrosine kinases at its C-terminus [10], leading CagA to bind and activate SHP2 and signal via ERK [11]. Importantly, CagA is also responsible for activating the signal transducer and activator of transcription 3 (STAT3) *in vitro* and *in vivo* [12], though this may not necessarily be dependent upon CagA phosphorylation [11].

STAT proteins are constitutively expressed in several neoplasms, including gastric, breast, head and neck, and prostate cancers [13]–[16]. Upon phosphorylation of the tyrosine 705 residue and acetylation at lysine 685, STAT3 dimerizes and enters the nucleus where it functions to transcriptionally regulate a wide array of genes [17], [18]. Constitutive activation of STAT3 protein has been shown to prevent apoptosis and increase cell proliferation and metastasis in a number of cancers, including gastric cancer [19], [20].

One of the hallmarks of gastric tumor progression is the acquisition of more invasive and migratory phenotypes during the epithelial-mesenchymal transition (EMT). During EMT, gastric epithelial cells undergo phenotypic changes characterized by the loss of cell adhesion molecules, particularly the epithelial cadherin (E-cadherin) [21]. The transcription factor Snail, a zinc-finger protein, has been characterized previously as an important regulator of EMT due to its activation via Nuclear Factor kappa Beta (NF-kB) [22] and subsequent repression of E-cadherin in epithelial tumor cells [23], [24]. Additionally, studies using gain-of-function and loss-of-function approaches have identified Snail as a repressor of RKIP transcription in metastatic prostate cancer cells [25].

RKIP is a member of the phosphatidylethanolamine-binding protein family and a negative regulator of the ERK1/2 (Extracellular Signal-Regulated Kinase) [26], NF-kB [27] and GRK (G Protein-Coupled Receptor Kinase) [28] pathways. RKIP thus plays an important role in regulating cell survival and apoptosis, in addition to potentiating the efficacy of chemotherapeutic agents [29]. RKIP has also been identified as a metastasis suppressor protein [30], and in gastric adenocarcinoma patients there exists a positive correlation between RKIP expression and patient survival and an inverse correlation between expression of RKIP and STAT3 [19]. RKIP expression and function can be regulated by post-translational modifications. For example, phosphorylation of RKIP by protein kinase C at serine-153 prevents RKIP’s ability to bind to its target molecule, thereby inactivating RKIP function [31]. Further, RKIP repression via promoter methylation can be overcome by methylation and histone deacetylase inhibitors [25].

Because of the important roles of RKIP, STAT3 and *H. pylori* in the pathogenesis of gastric cancer, we investigated whether *H. pylori* signals through RKIP. Our studies suggest that a complex interaction between *H. pylori’s cagPAI*, RKIP, STAT3, and Snail acts to dysregulate gastric epithelial cell apoptosis by modulating RKIP function, a mechanism that defines a central role for RKIP in *H. pylori*-associated gastric carcinogenesis.

## Materials and Methods

### Reagents

All reagents and chemicals were purchased from Sigma Chemical Co. (St. Louis, MO) unless otherwise noted. MG-132 was purchased from Calbiochem (Gibbstown, NJ) dissolved in DMSO and used at concentration of 10 mM. Interleukin-6 (IL-6) was purchased from BD Biosciences (San Diego, CA). Protein quantification reagents were obtained from Bio-Rad Laboratories, Inc. (Hercules, CA). Enhanced chemiluminescence reagents and secondary mouse and rabbit horse radish peroxidase-conjugated for Western blot analysis were from GE Healthcare (Piscataway, NJ). The actin-HRP, phosphorylated-RKIP (pRKIP) and STAT3 antibodies were purchased from Santa Cruz Biothechnology (Santa Cruz, CA). The antibodies to STAT3 pS727 and pY705 and PARP were purchased from Cell Signaling Technology (Beverly, MA) and the antibody to RKIP from Millipore, Billerica, MA. The antibody to Snail was purchased from Abcam (Cambridge, MA).

### Cells and Plasmids

The human gastric carcinoma cell line AGS (CRL-1739) was purchased from American Type Culture Collection (Manasas, VA). MKN28 cells were donated by Dr. Richard Peek, Vanderbilt University, Nashville, TN and were originally purchased from Riken Cell Bank, Ibaraki, Japan. The expression plasmids for pcDNA3, c-myc STAT3, CMV-HA-RKIP (HA-RKIP) and CMV-HA empty vector (EV) have been described [18], [26]. The RKIP S153V plasmid was provided by Dr. Marsha Rosner, University of Chicago, Chicago, IL.

### *H. pylori* Strains and Culture Conditions

Wild type *H. pylori* strains or isogenic *H. pylori* mutants were co-cultured with the AGS or MKN gastric cell lines as previously described [32] at a multiplicity of infection (MOI) of 100∶1 in all experiment unless otherwise stated.

### Transfection of AGS Cells

AGS cells were transiently transfected using the GenJet plasmid transfection reagent (Signagen Laboratories, Gaithersburg, MD) according to the manufacturer’s protocol for a 6-well plate format. Total DNA quantities of between 1 and 2 µg were transfected per sample. Transfection conditions were assessed and optimized by analysis of cells transfected with a Green Fluorescent Protein (GFP)-expressing RKIP plasmid. Transfection efficiencies were consistently in the range from 75–85%.

### Protein Extraction and Western Blot Analysis

Total cell extracts and subcellular fractionations were prepared and immunoblotted as previously described [29], [32]. Protein concentrations were determined using the BCA Protein Assay (Thermo Scientific). Densitometry of Western blots was performed according to the protocol listed at the following site: http://lukemiller.org/journal/2007/.

### Realtime PCR

Two µg of RNA was converted to cDNA using RevertAid First-Strand cDNA Synthesis Kit (Thermo Scientific). Quantitative real-time PCR was performed using 2× QIAgen QuantiFast SYBR Green I (Roche). The primers for Snail were forward: AGCTCTCTGAGGCCAAGGATCT, reverse: TGTGGCTTCGGATGTGCAT and beta-actin: forward: CTGGCACCACACCTTCTACAA, reverse: CAGCCTGGATAGCAACGTACA. The following typical profile times used were for 40 cycles: an initial step at 95°C for 10 min, followed by 95°C for 15 s and 60°C for 1 min. The relative expression level was calculated using the 2-ΔΔCT method as described previously [33].

### STAT3 and RKIP Luciferase Reporter Assays

Cells (2×10^5^ cells/well in 6-well plates) were transiently transfected with 0.1 µg (STAT3, RKIP) or 0.05 µg (NF-kB) of a reporter plasmid containing either the STAT3 binding SIE-fragment of the promoter region of the mouse IRF1 gene (p2xSIE-Luc) or the RKIP promoter region plus the indicated plasmids as previously described [18]. Approximately 24 h after transfection, cells were treated with the indicated drug or infected with *H. pylori* overnight or left untreated. The luciferase activity in the cytosolic supernatant was evaluated using the Luciferase Reporter Assay (Promega) and measured using a luminometer to estimate transcriptional activity [18].

### Apoptosis Assays

Apoptosis was quantified in separate assays by flow cytometry and DNA fragmentation ELISA. For flow cytometry, the percentage of apoptotic cells (sub-G_O_) was determined by flow cytometric analysis of propidium iodide stained cells [29]. Cytoplasmic histone-associated DNA fragmentation was measured with the Cell Death Detection ELISA Plus kit (Roche, Indianapolis, IN) according to the manufacturer’s instructions.

Cytoplasmic histone-associated DNA fragmentation was measured with the Cell Death Detection ELISA Plus kit (Roche, Indianapolis, IN) according to the manufacturer’s instructions. The experiments were repeated 3 times and performed in duplicate.

### Lentivirus-mediated Knockdown of RKIP

#### Lentivirus constructs

pLKO.1 puro-resistance lentiviral construct RHS3979-97070798 and RHS3979-98492779 were purchased from Open Biosystems (Huntsville, AL). The constructs contained a puromycin selection marker and were grown in Luria Broth containing ampicillin at 37°C. The broth was centrifuged at 10,000×*g* for 10 min. and the supernatant discarded. DNA from the pellets was purified using the QIAGEN Plasmid Plus Maxi Kit.

#### Lentivirus production

293T packaging cells were seeded in low-antibiotic growth media (DMEM, 10% heat inactivated FBS, 0.1× Penicillin/Strepomycin/Glutamine). Cells were incubated for 24 h (37°C, 5% CO_2_), or until they were approximately ∼70% confluent. The media on the 293T packaging cells was replaced with high growth media containing DMEM. A mixture of the transfection plasmids were prepared as follows: packaging plasmid (ΔVpr.89), envelope plasmid (VSV-G), hairpin-pLKO.1 and empty vector.

FUGENE transfection reagent was prepared in DMEM according to manufacturers instructions. Briefly, the 3 plasmids were added dropwise to the FUGENE and DMEM and mixed. The mixture was then allowed to incubate for 20–30 min. at room temperature. The transfection mix was then carefully added to the packaging cells. The cells were incubated for approximately 18 h. The transfection media was then discarded the following morning and replaced with high-growth media. Cells were incubated for 24 h. Lentivirus containing media was harvested containingand additional high-growth media added. Cells were incubated for 24 h and the media harvested. Typical collection was for 2–3 time points. All viral harvests were pooled.

#### Lentivirus infection of AGS cells

AGS cells were grown to approximately 50% confluence. The viral supernatants were added with polybrene. Twelve hours following infection, the viral media was discarded and replaced with viral media and incubated for an additional 12 h. The media was discarded and replaced with Ham’s F12 medium. Cells were incubated for 24 h. Cells were split into selection media containing of puromycin and allowed to incubate for 24 h.

### Statistical Methods

All cell culture experiments were repeated at least 3 times, unless indicated otherwise, and paired t-tests were used to determine statistical significance.

## Results

### *H. pylori* Infection Increases Phosphorylation of RKIP

**Figure 1:**
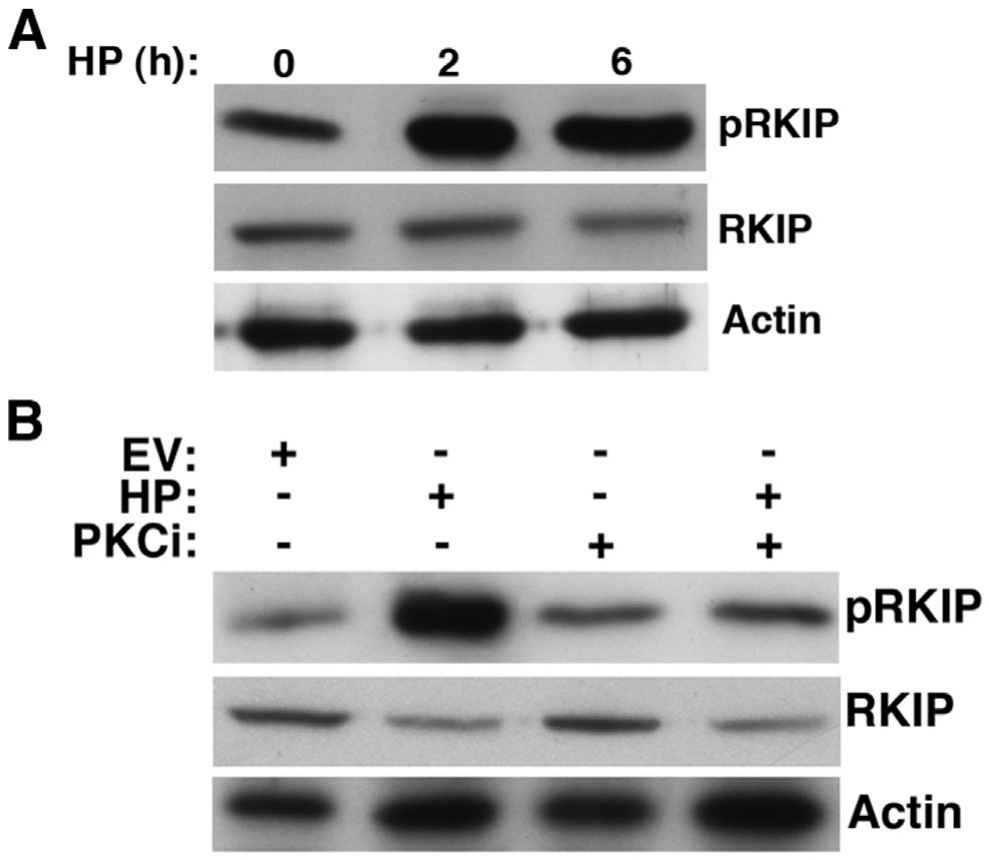
*H. pylori* infection results in RKIP phosphorylation. (A) Western blot analysis of AGS cells co-cultured with *H. pylori* (HP) at MOI of 100∶1 for 2 and 6 h and examined for pRKIP, RKIP, and actin expression. Densitometry was performed on three independent experiments and band intensities normalized in comparison to Actin for each time point. Our results indicated a 3.126 fold increase (average intensity 0.44 vs 1.376) of pRKIP after 2 h and 1.384 fold increase (average intensity 0.6774 vs 0.938) of RKIP after 2 h of *H. pylori* infection. (B) AGS cells co-cultured for 6 h in the presence or absence of the PKC inhibitor bisindolylmaleimide (Bis), were examined for the expression of pRKIP, RKIP and actin.All treatments were performed in 1% DMSO as a vehicle control (Bis).

RKIP inhibits several cell survival pathways, including those mediated through NF-kB and Jak/STAT [22]. To elucidate the effect of *H. pylori* infection on RKIP in gastric cells, AGS cells were infected with *H. pylori* and harvested 2 h and 6 h later. As shown in Fig. 1A, levels of phosphorylated RKIP (pRKIP) were elevated after 2 h (3.126-fold) and 6 h (2.9-fold) after *H. pylori* infection whereas total RKIP protein expression increased 1.4-fold after 2 h and 1.75-fold after 6 h of *H. pylori* infection. Similar results were also obtained with MKN gastric cancer cells (see Figure S1).

### *H. pylori* Induced Phosphorylation of RKIP is PKC-dependent

The phosphorylation of RKIP on serine 153 by protein kinase C (PKC) abrogates its ability to bind to Raf and inhibit downstream MAP kinase signaling [31]. We examined whether phosphorylation of RKIP by *H. pylori* was PKC-dependent. AGS cells were infected with *H. pylori* for 6 h, in the presence or absence of 40 µM bisindolylmaleimide (Bis, a PKC inhibitor). Our results indicate that the levels of phosphorylated RKIP was inhibited 3.9-fold and RKIP 1.36-fold after *H. pylori* infection in the presence of the PKC inhibitor, suggesting that RKIP phosphorylation by *H. pylori* involves, but may not be entirely dependent upon, the PKC-regulated pathway (Fig. 1B).

### IL-6 Induces Phosphorylation of RKIP and *H. pylori-*activates STAT3

**Figure 2:**
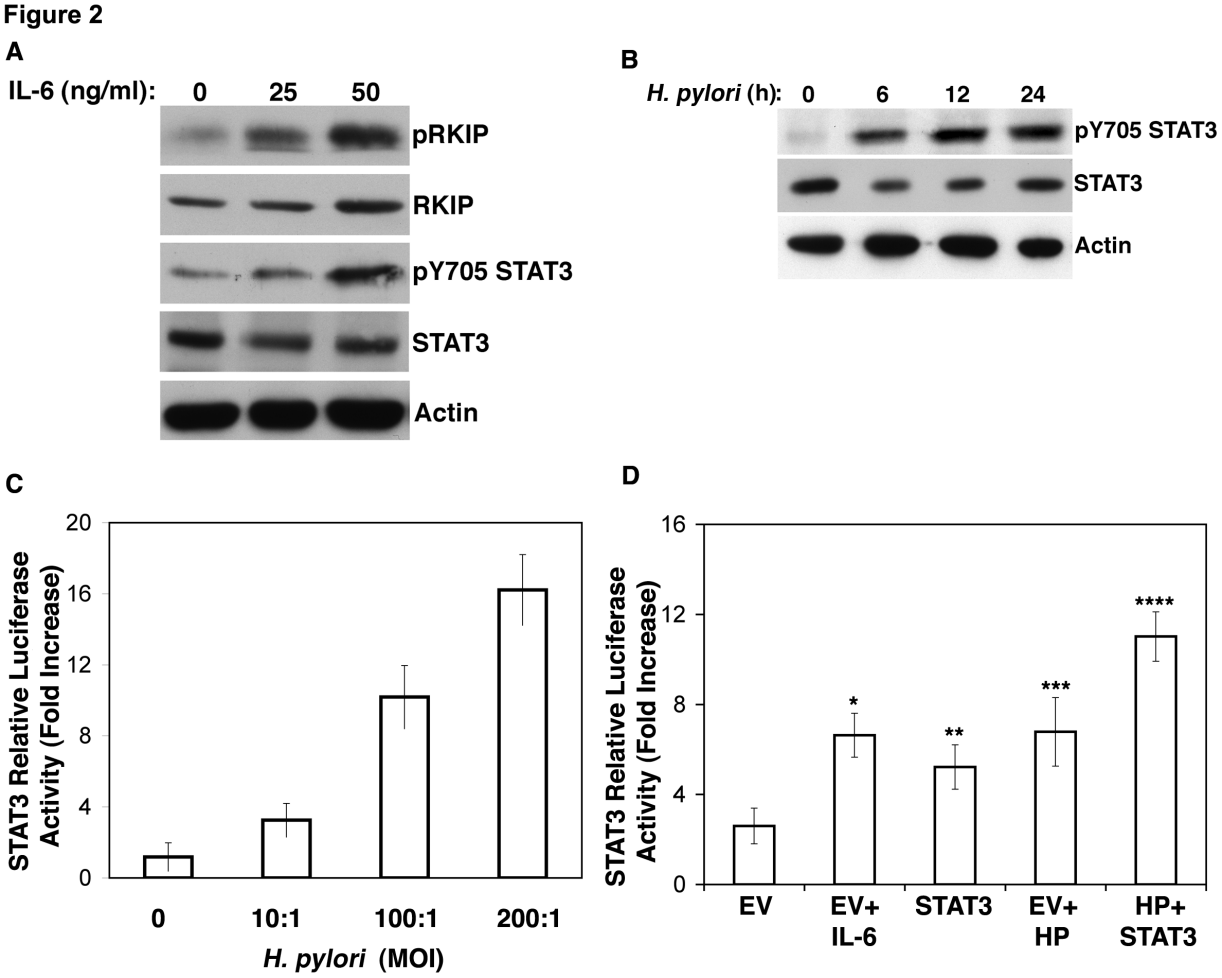
IL-6 promotes RKIP and STAT 3 transcription and phosphorylation; *H. pylori* infection induces STAT3 transcription and phosphorylation. (A) Western blot analysis of AGS cells treated with the indicated concentrations of IL-6 and for 6 hours. Densitometry was performed and for pRKIP expression, our results indicate a 1.8 fold incerase of pRKIP (average intensity 0.59 vs 1.051) in cells treated with 25 ng/ml IL-6 and a 1.35 fold increase (average intensity 0.59 vs 0.772) in cells treated with 50 ng/ml IL-6. For RKIP expression, our results indicate a 0.8 fold incerase of pRKIP (average intensity 0.69 vs 0.563) in cells treated with 25 ng/ml IL-6 and a 1.05 fold increase (average intensity 0.69 vs 0.745) in cells treated with 50 ng/ml IL-6 when normalized to actin at each time point. (B) Western blot analysis of AGS co-cultured with *H. pylori* and examined for pY705 STAT3 for the indicated times; (C) STAT3 luciferase reporter transcriptional assay of AGS cells co-cultured with *H. pylori* at the indicated MOI; (D) STAT3 luciferase reporter assay of AGS cells transiently transfected with STAT3 for 24 h and co-cultured with *H. pylori* and/or treated with IL-6 for 6 h. A paired t-test was performed to analyze the increase or decrease in STAT3 transcription of experimental samples when compared to empty vector (EV): *IL-6, p<0.0003; **STAT3, p<0.009; *** *H. pylori* p<0.0005; **** STAT3 and *H. pylori* p<0.0000023.

Since there is an inverse relationship between RKIP and STAT3 expression in gastric cancer specimens [19], we evaluated whether STAT3 and its key regulator IL-6 [17] affects pRKIP expression. IL-6 treatment at a dose of 25 or 50 ng/ml increased levels of pRKIP protein 1.8 and 1.35 fold, respectively and total RKIP protein expression decreased 0.8 and increased 1.05 fold, respectively (Fig. 2A). The phosphorylation of RKIP in response to IL-6 was PKC-dependent (data not shown). These data indicate that IL-6 can also induce phosphorylation of RKIP in gastric cells that may occur, in part, to a PKC-dependent pathway.

Macrophages release cytokines, including IL-6 during *H. pylori* infection [34] leading to STAT3 activation [17]. To investigate the effects of *H. pylori* infection on the activation of STAT3, AGS cells were transiently transfected with an IRF-1 reporter construct [18] and co-cultured with *H. pylori* at the indicated range of multiplicity of infection (MOI) for 24 h. Our results showed that at a MOI between 10–200∶1, *H. pylori* was able to induce STAT3 transcription (Fig. 2C) and STAT3 pY705 phosphorylation (Fig. 2B) within 6 h of infection. We next determined whether IL-6 could also stimulate STAT3 transcription in AGS cells. AGS cells were transiently transfected with IRF-1 and with EV and or c-myc-tagged STAT3 and then after 24 h cells treated with either IL-6 (50 ng/ml) or co-cultured with *H. pylori* at MOI of 100∶1. The results, depicted in Fig. 2D, demonstrate that IL-6 (p<0.0003) and *H. pylori* (p<0.0005) were each able to significantly stimulate STAT3 transcription, an effect that was enhanced when AGS cells were transfected with STAT3 and infected with *H. pylori* (p<0.0000023). The enhancement of STAT3 activation was significantly increased when AGS cells were co-treated with IL-6 and *H. pylori* when compared to treatment with IL-6 (p<0.000028) or *H. pylori* (p<0.0003) alone.

### Phosphorylated RKIP Induces its Own Transcription

**Figure 3:**
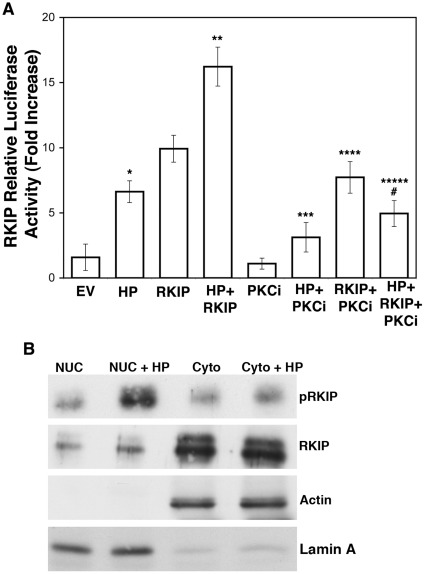
*H. pylori* infection results in increased RKIP transcription and nuclear localization. (A) RKIP transcription reporter assay of AGS cells transiently transfected with RKIP luciferase construct and HA-RKIP for 24 h, then co-cultured with *H. pylori* for 12 h in the presence or absence of the PKC inhibitor. In comparison to empty vector controls, relative transcriptional activity was significantly increased for * *H. pylori*, p<0.002; and ** *H. pylori*+RKIP, P<0.0003. Comparing the loss of relative luciferase activity of *H. pylori* and RKIP when compared to *H. pylori*, RKIP and Bis, p<0.0004. Data represents the mean +/− standard deviation (sd) of the fold increase relative to empty vector controls in 2 independent experiments performed in duplicate. (B) Western blot analysis of nuclear and cytosolic fractions of AGS cells co-cultured with *H. pylori* for 4 h for the expression of pRKIP and total RKIP. Actin and laminA provide verification of successful cytoplasmic and nuclear fraction separation.

We used an RKIP luciferase reporter assay to investigate the effects of *H. pylori* infection on RKIP transcriptional activity. *H. pylori* significantly increased RKIP transcription (p<0.002) with a greater than 10-fold increase occurring with RKIP overexpression and a greater than 16-fold increase with the combination of *H. pylori* and RKIP (p<0.0003) (Fig. 3A) when compared to untreated AGS cells transfected with empty vector. There was a significant increase (p<0.0001) in RKIP transcription with *H. pylori* infection and RKIP overexpression when compared to cells transfected with RKIP without infection (Fig. 3A). We repeated these experiments in the presence of Bis to inhibit PKC activity to determine the increase in RKIP transcription was due to phosphorylation. In the presence of the PKC inhibitor, *H. pylori* increased RKIP transcription and RKIP overexpression also resulted in the enhancement of RKIP promoter activity. Bis diminished RKIP transcription induced by RKIP overexpression and *H. pylori* infection greater than 4-fold, when compared to cells in with RKIP overexpression and suggests that these effects were dependent upon RKIP phosphorylation (Fig. 3A). We examined the localization of RKIP after *H. pylori* infection. Immunoblotting subcellular AGS cells fractions demonstrated that pRKIP is localized to the nucleus while RKIP remains mainly in the cytosol after infection, (Fig. 3B). Together, these data imply that *H. pylori* may promote the translocation of pRKIP into the nucleus where it can activate RKIP transcription.

### *H. pylori*-induced RKIP Phosphorylation Depends on *H. pylori’s cag* Pathogenicity Island and RKIP Serine 153

**Figure 4:**
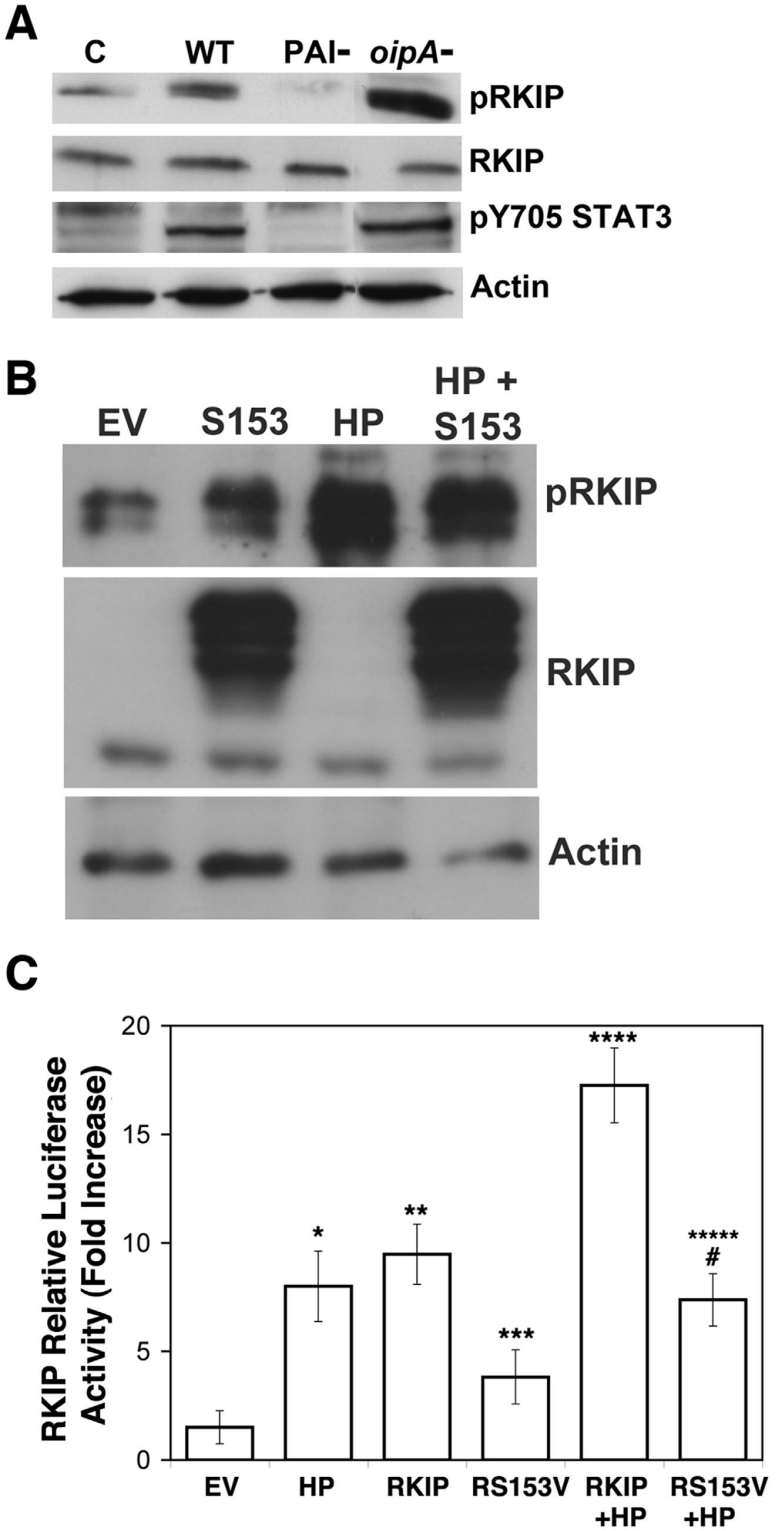
The pathogenicity island of *H. pylori* is responsible for RKIP phosphorylation. Western blot analysis of (A) AGS cells co-cultured with *H. pylori* strains for 6 h and examined for the indicated proteins. C = control (uninfected), WT = AGS cells infected with wild type *H. pylori* for 6 h, *PAI*- and *oipA*- represent isogenic mutants lacking these genes. (B) AGS cells transiently transfected for 24 h with RKIP S153 cDNA or 24 h and co-cultured with *H. pylori* for 6 h. (C) RKIP luciferase reporter assay of AGS cells transiently transfected with S153V RKIP in the presence or absence of *H. pylori* infection. In comparison to empty vector controls, the relative activity of RKIP transcription was increased by: *H. pylori, p<0.002; **RKIP, p<0.002; ***S153V, p<0.03, ****H. pylori and RKIP, p<0.0005; *****H. pylori and S153V, p<0.003. **, RKIP transcriptional activity was significantly decreased by the S153V compared with the wild type RKIP construct in response to *H. pylori, #*, p<0.0003. The data represents the mean +/− sd of 2 independent experiments performed in duplicate.

To evaluate the role of specific *H. pylori* factors in the phosphorylation of RKIP, wild type *H. pylori* and isogenic mutants lacking the entire cag *PAI or* the *oipA* gene were co-cultured with AGS cells for 6 h. The *H. pylori* mutant lacking the cag*PAI* was unable to induce RKIP phosphorylation, whereas the wild type stain and the *oipA* mutant strongly induced RKIP phosphorylation (Fig. 4A). The same trend was observed on STAT3 pY705. These results suggest that genes within *H. pylori’s* cagPAI are necessary for the induction of RKIP and STAT3 phosphorylation.

To investigate if mutation of serine 153 affects *H. pylori*-mediated RKIP phosphorylation and transcriptional activation, AGS cells were transiently transfected with an RKIP construct in which serine was substituted with valine at position 153 (S153V) and then co-cultured with *H. pylori*. Once again we observed a greater then 16-fold increase in RKIP promoter activity in cells with RKIP overexpression and *H. pylori* infection (p<0.0005). However, in cells transfected with RKIP S153V before *H. pylori* infection, there was a 2.5-fold reduction in transcriptional activity (p<0.0003) when compared to the *H. pylori* infection in wild type RKIP overexpressing cells (Fig. 4C). In addition, overexpression of S153V RKIP inhibited *H. pylori*-mediated RKIP phosphorylation (Fig. 4B). Taken together, these results indicate that the phosphorylation and transcriptional activation of RKIP is dependent upon *H. pylori’s* cagPAI and also upon phosphorylation of RKIP at S153.

### *H. pylori* Infection Results in RKIP Degradation and the Induction of Snail

**Figure 5:**
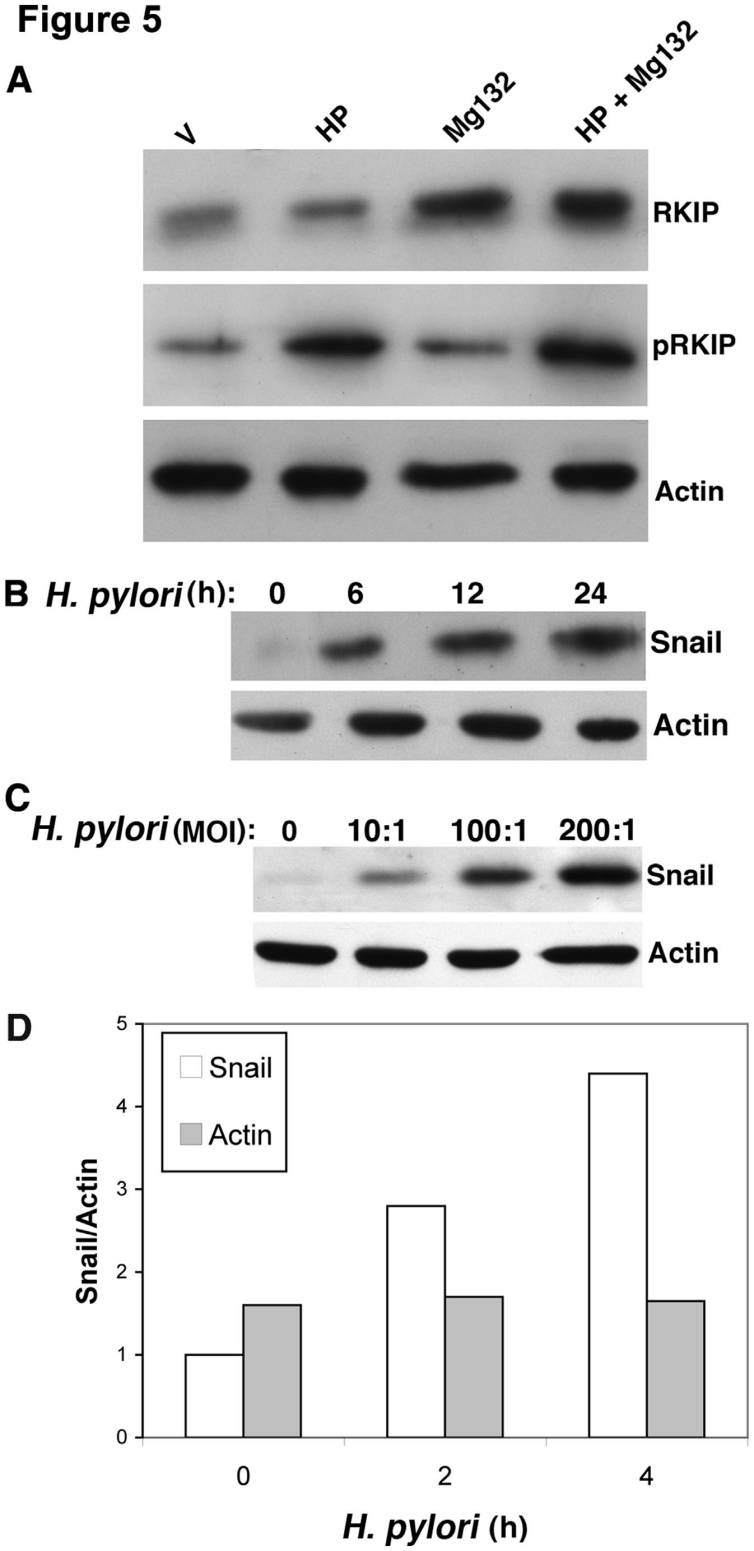
*H. pylori* targets RKIP for proteasomal degradation and results in the induction of Snail. AGS cells were (A) treated with MG132 and examined for the indicated proteins via Western blot analysis. V represents vehicle (DMSO) control. (B, C) Cells were co-cultured with *H. pylori* for the indicated times and examined for the expression of Snail or (D) measured for Snail mRNA by real-time PCR at the indicated times after *H. pylori* infection.

Because increased RKIP transcription induced by *H. pylori* infection was not associated with increased steady state total RKIP protein expression, we examined whether *H. pylori* might simultaneously increase the rate of degradation of RKIP protein through proteasome-mediated degradation, as previously suggested [35]. MG132 increased RKIP protein levels in the presence or absence of *H. pylori* infection, consistent with *H. pylori* increasing proteasomal RKIP degradation (Fig. 5A).

**Figure 6:**
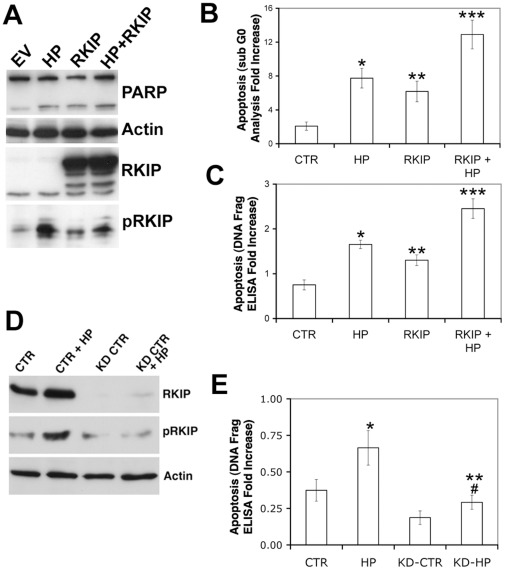
RKIP enhances *H. pylori* mediated apoptosis. (A) Western blot analysis of AGS transiently transfected with RKIP for 24 h then infected with *H. pylori* for 6 h. Densitometry analysis for the average of 2 independent experiments indicated a 1.53 fold increase in apoptosis (average intensity of 0.19 vs 0.295) in *H. pylori* infected cells; 2.1 fold increase (average intensity of 0.19 vs 0.403) in cells transfected with RKIP; a 2.6 fold increase (average intensity of 0.19 vs 0.495) in cells infected with *H. pylori* and transiently transfected with RKIP when normalized to actin. In parallel apoptosis was measured by (B) flow cytometry. C) An ELISA based DNA fragmentation assay was used to measure apoptosis. Compared to empty vector control in (B) apoptosis was increased by * H. pylori, p<0.0008; ** RKIP, p<0.003; *** *H. pylori* and RKIP, p<0.0005. In (C) compared to empty vector controls, apoptosis was significantly increased by: * *H. pylori*, p<0.000063; ** RKIP, p<0.006; *** H. pylori and RKIP, p<0.0007. The data for B and C represents the mean +/− sd of 2 independent experiments performed in duplicate. (D) Western blot analysis of AGS cells infected with Lentivirus to knock down RKIP expression prior to 6 h infection with *H. pylori*. CTR =  uninfected AGS cells, HP =  AGS cells infected with *H. pylori*, KD =  AGS cells infected with lentivirus to knockdown RKIP, KD+RKIP AGS cells infected with lentivirus to knockdown RKIP and infected with *H. pylori* for 16 h. (E) Apoptosis (by DNA fragmentation ELISA) was measured after 16 h of *H. pylori* infection. Apoptosis was significantly increased by *H. pylori* in AGS cells *p<0.0007; and in AGS cells with RKIP knockdown, **p<0.003 and was decreased comparing *H. pylori*-infected RKIP knockdown AGS cells with *H. pylori*-infected parental AGS cells, #p<0.0006. The data shown represents the mean +/− sd of 2 experiments performed in triplicate.

Another mechanism that could account for the lack of change in RKIP protein or mRNA expression would be transcriptional repression of RKIP after *H. pylori* infection. Snail is a transcription factor that plays an important role in EMT [23] as well as being a known transcriptional repressor of RKIP in prostate cancer cells [25]. To investigate the effects of *H. pylori* infection on the expression of Snail and RKIP, AGS cells were co-cultured with *H. pylori* at a MOI of 100. Snail mRNA expression was strongly induced after 2–4 h of infection (Fig. 5D) Western blot analysis indicated that *H. pylori* infection resulted in a time and dose dependent increase in the Snail protein levels (Fig. 5B/C). This result is not consistent with our data on RKIP transcription after *H. pylori* infection (Fig. 3) and suggests that *H. pylori* infection may in the induction of protein(s) that would abrogate the effect of Snail on RKIP transcription. We are currently investigating this possibility by Mass Spectometry analysis using parental and RKIP knockdown cells (Fig. 6).

### RKIP Enhances *H. pylori*-mediated Apoptosis

*H. pylori* induces gastric epithelial cell apoptosis [8]. Since RKIP can promote apoptosis [29], we examined if the induction of pRKIP after *H. pylori* infection, could be responsible for *H. pylori*-induced apoptosis. AGS cells were transiently transfected with RKIP or an empty vector, infected with *H. pylori* for 16 h and apoptosis evaluated via PARP cleavage flow cytometry and DNA fragmentation. In some experiments RKIP was inhibited by lentivirus-mediated RKIP knockdown. As shown in Fig. 6A, *H*. *pylori* induced the cleavage of PARP, an effect that was increased by ectopic expression of RKIP. Flow cytometry analysis indicated that *H. pylori* infection resulted in an approximately 4-fold increase in apoptosis (p<0.0008), RKIP overexpression, a 3-fold increase (p<0.003) and the combination a 6-fold increase (p<0.0005) when compared to untreated AGS cells (Fig. 6B). In the ELISA-based DNA fragmentation assay, apoptotic activity increased: 2.5 fold (p<0.000063) in cells infected with *H. pylori*; 1.8 fold (p<0.006) in cells transiently transfected with RKIP; and 3.5 fold (p<0.0007) with the combination (Fig. 6C). To determine whether RKIP was responsible for *H. pylori*-mediated apoptosis, we suppressed RKIP expression using lentivirus-mediated RNA inhibition and observed a reduction in RKIP protein levels by Western blot analysis demonstrating the reduction of RKIP in untreated and H. pylori infected AGS cells (Fig. 6D). In our DNA fragmentation analysis, in parental AGS cells *H. pylori* infection resulted in a 2-fold increase (p<0.0007) in apoptosis (Fig. 6E). In the RKIP knockdown AGS cells, *H. pylori* infection resulted in a 1.5-fold increase in apoptosis (p<0.003) (Fig. 6E). The reduction in apoptosis between parental and RKIP knockdown AGS cells was statistically significant (p<0.0006). These results indicate that RKIP is necessary for *H. pylori*-mediated apoptosis.

## Discussion

Chronic gastritis and altered cellular turnover induced by *H. pylori* infection promote the development of distal gastric adenocarcinoma [36]. *H. pylori* can regulate gastric epithelial apoptosis through several mechanisms. For example, following infection and adherence to gastric epithelial cells, the cag secretion system serves to alter intracellular signal transduction resulting in the activation of NF-kB. NF-kB can translocates to the nucleus to activate transcription of pro-apoptotic genes [37]. *H. pylori* can also induce apoptosis by increasing expression of FAS and its ligand (FASL) leading to the activation of the extrinsic apoptosis pathway [38]. Paradoxically, *H. pylori* may also activate pathways that downregulate apoptosis [39], especially late in the course of chronic infection [40]. This adaptive response of epithelial cells to resist apoptosis in chronic *H. pylori* infection may contribute to *H. pylori*-induced gastric carcinogenesis [41]. The apoptotic response of gastric epithelial cells to *H. pylori* is also dependent upon strain-specific virulence factors. For example, infection with cag PAI-positive strains may induce apoptosis more rapidly than cag PAI-negative strains [42]. The *H. pylori vacA* gene product stimulates the intrinsic apoptotic pathway leading to the mitochondrial release of cytochrome c, and caspase-3 activation [43]. VacA-induced apoptosis is associated with a reduction of STAT3 leading to the downregulation of Bcl-2 and Bcl-X_L_ [43]. In another study, it was demonstrated that *H. pylori* induces apoptosis by a pathway involving the sequential induction of apical caspase-8 activity, the pro-apoptotic proteins Bad and Bid, caspase-9 activity, and effector caspase-3 activity [44].

Our study describes another mechanism by which *H. pylori* infection can promote apoptosis in gastric cancer cells, specifically by promoting RKIP phosphorylation. The ability of RKIP to inhibit Raf/MAPK signaling [26], [27] and promote apoptosis has been well documented [29]. The interaction of theses pathways and RKIP expression levels has been implicated at many steps of tumor formation and/or progression [30]. Furthermore, overexpression of RKIP results in the inhibition of metastasis and invasiveness in various tumor models [45]–[48]. The underlying mechanism of the differential expression of pRKIP and RKIP is not known. We had expected that relatively higher levels of pRKIP after infection might correlate with lower RKIP levels. However, we found that *H. pylori* infection resulted in the degradation of RKIP protein, possibly allowing MAPK signaling and apoptosis induction in gastric cancer after *H. pylori* infection. PKC-mediated RKIP phosphorylation can disrupt the ability of RKIP to bind to Raf and inhibit MAPK signaling [31], however, there have not previously been any reports on the role of pRKIP in the regulation of apoptosis. Previous studies from our laboratory have shown that RKIP overexpression results in the direct activation of pro-caspase 8 [29]. Although phosphoryation results in nuclear relocalization, followed by RKIP activation of its own transcription, the levels of RKIP protein do not increase. This suggests a different mechanism of RKIP regulation than what has been previously reported. We are currently examining the mechanism by which pRKIP triggers apoptosis in gastric cancer cells after *H. pylori* infection.

Cag-positive *H. pylori* significantly upregulate the EMT-associated genes Snail, Slug and vimentin in association with the induction of MMP-7, suggesting a role for these proteins in gastric cancer development [49]. In our study we observed the rapid induction of pRKIP protein after *H. pylori* infection, and an increase in RKIP transcription, and an upregulation of Snail mRNA and protein expression. Although Snail was identified as a transcriptional repressor of RKIP [25], our study indicates that it probably has no effect on the phosphorylated form of RKIP, since after infection we did not observe a repression of RKIP transcription.

Infections with cagPAI-possessing strains of *H. pylori* are associated with a stronger inflammatory response in the stomach and pose a greater risk of developing peptic ulcers or stomach cancer than strains lacking the cag island [36]. *H. pylori* induces an intense inflammatory response and locally high levels of several cytokines including interleukin 6 (IL-6) [34]. Treatment of AGS cells with IL-6 led to the phosphorylation of RKIP, suggesting that in addition to promoting apoptosis, pRKIP may also be involved in the inflammatory response to *H. pylori* infection. The relationship between RKIP, apoptosis, inflammation and the signal transduction pathways activated by *H. pylori* await further dissection, as does the precise role of RKIP in *H. pylori*-mediated gastric carcinogenesis. Further analysis is warranted, including utilizing a RKIP transgenic knockout model [50] to accurately define the role of RKIP in *H. pylori*-mediated gastric cancer progression.

## Supporting Information

Figure S1 *H. pylori* infection results in RKIP phosphorylation and transcriptional activation in MKN28 cells. (A) MKN26 cells were infected with H. pylori (MOI 200∶1) in the presence or absence of the PKC inhibitor bisindolylmaleimide for 6 h and measured for the expression of pRKIP, RKIP and actin. (B) H. pylori infection results in the transcripitional activation of RKIP in MKN28 and AGS cells. MKN28 and AGS cells were transiently transfected with RKIP luciferase construct and HA-RKIP for 24 h, then co-cultured with H. pylori for 12 h. Data represents the mean +/− standard deviation (sd) of the fold increase relative to empty vector controls in 2 independent experiments performed in duplicate.
(TIF)
