# Supplementary figures and images for: Regulation of RKIP Function by Helicobacter pylori in Gastric Cancer
Source: PLoS One. 2012 May 25;7(5):e37819. doi: 10.1371/journal.pone.0037819 (PMC3360604; doi:10.1371/journal.pone.0037819)

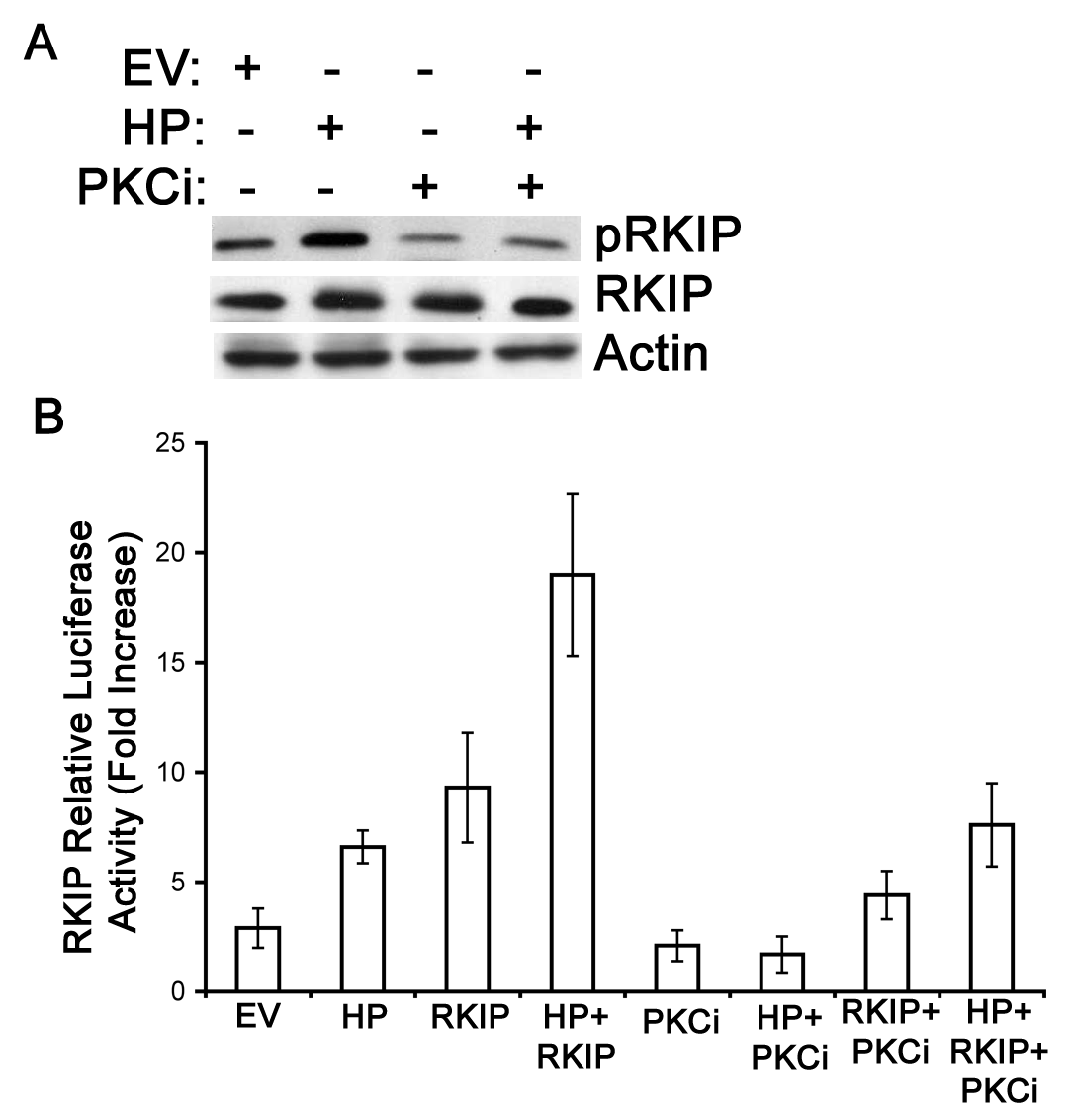

Supplement: Figure S1 — H. pylori infection results in RKIP phosphorylation and transcriptional activation in MKN28 cells. (A) MKN26 cells were infected with H. pylori (MOI 200∶1) in the presence or absence of the PKC inhibitor bisindolylmaleimide for 6 h and measured for the expression of pRKIP, RKIP and actin. (B) H. pylori infection results in the transcripitional activation of RKIP in MKN28 and AGS cells. MKN28 and AGS cells were transiently transfected with RKIP luciferase construct and HA-RKIP for 24 h, then co-cultured with H. pylori for 12 h. Data represents the mean +/− standard deviation (sd) of the fold increase relative to empty vector controls in 2 independent experiments performed in duplicate. (TIF) [file pone.0037819.s001.tif]
